# Rearing *Tenebrio molitor* and *Alphitobius diaperinus* Larvae on Seed Cleaning Process Byproducts

**DOI:** 10.3390/insects12040293

**Published:** 2021-03-27

**Authors:** Christos I. Rumbos, Dimitrios Bliamplias, Marina Gourgouta, Vasilios Michail, Christos G. Athanassiou

**Affiliations:** 1 Laboratory of Entomology and Agricultural Zoology, Department of Agriculture, Crop Production and Rural Environment, University of Thessaly, 38446 Volos, Greece; mdimitris1997@gmail.com (D.B.); magkourg@agr.uth.gr (M.G.); athanassiou@uth.gr (C.G.A.); 2 Fyto-Animal Services (F.A.S.), 40009 Larissa, Greece; mihailva@otenet.gr

**Keywords:** circular economy, edible insects, feed conversion efficiency, insect farming, insects as food and feed, organic side-streams

## Abstract

**Simple Summary:**

Insects have recently attracted considerable scientific and commercial interest as an alternative nutrient source. Agricultural wastes is a big, often untapped, pool of nutrients which could be used for insect rearing. Insects can actually feed on these byproducts, converting these low-cost materials to insect protein that will be further exploited as food or feed. In this study, we evaluated ten byproducts of the seed cleaning process of cereals and legumes as feed for larvae of two insect species, i.e., the yellow and the lesser mealworm. The larval growth and survival, as well as the time it took the larvae to become pupae and the amount of feed consumed by larvae were monitored throughout the experiments. According to our results, most of the byproducts tested supported the larval growth of both species. However, larvae grew better when fed with lupin and triticale byproducts. These results aim to enhance the sustainability profile of insect production and integrate insect farming with circular economy practices.

**Abstract:**

The exploitation of agricultural byproducts and organic side-streams as insect feeding substrates is advantageous for insect farming both from an economic and a sustainability perspective. In this context, in the present study we evaluated the suitability of ten byproducts of the cereal and legume seed cleaning process for the rearing of larvae of the yellow mealworm, *Tenebrio molitor*, and the lesser mealworm, *Alphitobius diaperinus*. Byproducts were offered singly to 20 *T. molitor* and 50 *A. diaperinus* larvae with provision of carrots as moisture source. After four weeks of undisturbed development, larval weight and survival was evaluated biweekly until pupation. Feed utilization and economic feasibility parameters were determined for each byproduct at the end of the bioassays. Our results show the suitability of several of the byproducts tested for the rearing of *T. molitor* and *A. diaperinus* larvae. The best results though among the byproducts tested in terms of larval growth and survival, development time and feed utilization were obtained with larvae fed with lupin and triticale byproducts, which efficiently supported complete larval development. The results of our study aim to boost the integration of circular economy strategies with insect farming practices.

## 1. Introduction

Agricultural wastes represent a very large reservoir of underrated resources, occurring during the production, processing and consumption of agricultural products [1]. Indicatively, around 18.4 billion tonnes of agricultural wastes, co-products and byproducts were produced in EU28 between 2010 and 2016 [1]. Insects can act as “bioreactors”, converting low-cost organic side-streams to insect protein [2,3]. Recently, the European association representing the stakeholders involved in insect production identified the evaluation of new, alternative feeding substrates for mass-produced insects as one of the major research priorities of the European insect sector [4]. The exploitation of agricultural wastes as insect feed substrates can offer both economic and environmental benefits to the insect sector. Among the other costs (labour, infrastructure, etc.), the cost of insect feedstock greatly contributes to the overall insect production cost [5], therefore, the use of low or zero economic value substrates as insect feedstocks is a feasible means to alleviate the overall production cost [6] and subsequently reduce the high insect meal market price [7,8]. Moreover, the valorisation of biowastes as insect feedstocks can strengthen the sustainability profile of insect farming [9] and is completely aligned with circular economy strategies that are persistently promoted in the European Union [10,11].

The yellow mealworm, *Tenebrio molitor* L. (Coleoptera: Tenebrionidae), and the lesser mealworm, *Alphitobius diaperinus* (Panzer) (Colepotera: Tenebrionidae), are two insect species that have attracted a lot of scientific and commercial attention in the last decade. Both species are approved as aquafeed ingredient in EU [12], whereas *T. molitor* was the first insect species to acquire an EU-wide approval for human consumption [13]. The upcycling and bioconversion of several organic side-streams and wastes by *T. molitor* has been studied by several studies [14,15,16,17,18,19,20,21,22,23,24]. For instance, an olive pomace-enriched substrate up to 25% enrichment sufficiently supported *T. molitor* larval growth, providing a means for exploitation, as well as management, of the olive processing byproducts [22]. Similarly, maize stover has also been proposed as a suitable dietary component for *T. molitor* larvae [19]. In the case of *A. diaperinus*, studies on the dietary inclusion of organic waste materials are rather limited [24,25,26]. *Alphitobius diaperinus* larvae can grow successfully in diets composed by several side-stream materials, such as spent grains and beer yeast, bread and cookie remains, potato steam peelings, and maize distillers’ dried grains with solubles (DDGS), the diets with high percentage of yeast-derived protein being more favourable in terms of larval survival, speed of development and growth [24]. In the same context, diets containing wheat middlings and rapeseed meal were shown to support good larval growth and development [25], providing larvae with a good nutrient profile [26].

Based on the above, the objective of the present study was to evaluate the suitability of ten agricultural byproducts, originating from the cereal (triticale, barley, durum wheat and oat) and legume (vetch, pea, lupin, lentil, lucerne and broad bean) seed cleaning process, as feeding substrates for *T. molitor* and *A. diaperinus* larvae.

## 2. Materials and Methods

### 2.1. Insects

Both species were reared at the Laboratory of Entomology and Agricultural Zoology (Volos, Magnesia, Greece) at 26 ± 1 °C, 55% relative humidity (r.h.) and continuous darkness. A diet consisted of wheat bran (90%) and dry instant yeast (10%) (Angel Yeast Co. Ltd., Yichang, China) and supplemented with fresh potato slices twice a week was used for both species as feeding substrate during lab rearing. Newly hatched larvae (<2 days (d) old) were used in the bioassays. To acquire newly hatched larvae, adults of *T. molitor* and *A. diaperinus* were left to oviposit for one week in white wheat flour. After this interval, adults were removed and eggs were collected through hand sieving with a 0.5-mm opening sieve. Larvae for experimentation were collected 2 d after the first larva hatched.

### 2.2. Byproducts

The byproducts tested derived from the seed cleaning process of major cereals (triticale, barley, durum wheat and oat) and legumes (vetch, pea, lupin, lentil, lucerne and broad bean) (Table 1). Drying in an oven at 105 °C to constant weight was performed to determine the dry matter of the byproducts. The crude protein content was determined by Kjeldahl analyses (N × 6.25; Behr Labor-Technik GmbH, Düsseldorf, Germany, K12-block standard digestion system, programmable infrared digestion device, S4 distillation unit) [27]. All byproducts, as well as information regarding their cost, were provided by a seed cleaning facility (Fyto-Animal Services (F.A.S., Larissa, Greece)).

### 2.3. Experimental Design

Plastic cylindrical vials (7.5 cm in diameter, 8.8 cm in height) were the experimental units for the bioassays. All byproducts were tested singly. One g of each byproduct was placed in each vial, using different vials for each substrate. A mixture of wheat bran and yeast (9:1) served as control in the bioassays. Before the beginning of the experiments, all byproducts were left at ambient conditions (26 ± 1 °C, 55 ± 5% r.h.) for 7 d, to equilibrate with the relative humidity level. All byproducts were ground (Thermomix TM31-1C, Vorwerk Elektrowerke GmbH and Co. K, Wuppertal, Germany) and hand-sieved with a 0.5-mm opening sieve. Groups of 20 *T. molitor* and 50 *A. diaperinus* early-instar larvae were created, weighted and transferred to the vials. Each treatment was replicated six times. Larvae were allowed to feed undisturbed *ad libitum* for a four-week period. Vials were monitored three times per week for food consumption to ensure that larvae will not run out of food. If food was totally consumed, new food was weighed, added, and recorded. As a moisture source, carrot slices (0.6 ± 0.1 g) were provided to larvae three times per week, with old carrot pieces being removed. At the end of the four-week period, larvae from each vial were separated from the feeding substrate, and larval survival and larval weight as a group were recorded. The same procedure was repeated every two weeks until the emergence of the first pupa. The development time was calculated, as the number of days between the start of the experiment and the day each vial was harvested. Food consumption and weight gained data were used to calculate the Feed Conversion Ratio (FCR, Equation (1)), i.e., the amount of feed needed (in kg) to obtain one kg of weight increase of the production animal

FCR = (Feed consumed)/(Weight gained)(1)

as previously described [28]. Additionally, the Specific Growth Rate (SGR, % day^−1^, Equation (2)) was calculated according to the following formula:

SGR = 100 × ((lnFBW − lnIBW))/days(2)

where FBW and IBW stand for final and initial body weight, respectively. Both FCR and SGR were calculated on fresh weight basis. For all calculations we made the assumption that all provided feed was consumed, whereas the weight of provided carrots was excluded from the calculations. Based on FCR, as well as on the price of each byproduct (Table 1), the Economic Conversion Ratios (ECR, € kg^−1^, Equation (3)) of the use of each byproduct for the rearing of *T. molitor* and *A. diaperinus* larvae were estimated using the following equation:

ECR = FCR × Diet Cost(3)

where FCR is expressed as kg diet kg^−1^ larvae and diet cost as € kg^−1^ diet, as previously described [29].

### 2.4. Statistical Analysis

The Kruskal–Wallis H test was performed to determine significant differences between treatments (*p* < 0.05) for FCR, SRG and ECR, followed by Dunn multiple comparisons for post-hoc testing. The Kaplan–Meier method was used to analyse the development time and a Mantel–Cox test was used to detect differences between treatments. Correlations between development time and FCR and SGR, as well as between FCR and ECR, were determined by Pearson correlation tests. Statistical analysis for all data was done using SPSS 26.0 (IBM Corporation, Armonk, NY, USA).

## 3. Results

The protein content of the byproducts ranged from 8.5 to 33.5%, the highest protein content being recorded for lupin and the lowest for triticale byproduct. As expected, most of the legume byproducts were rich in protein (20.8–33.5%), with the exception of lucerne byproduct (13.3%). In contrast, the cereal byproducts tested contained lower protein content (8.5–12.3%). All byproducts had a similar dry matter content (DM) (90.4–95.7%).

### 3.1. Tenebrio molitor

**Figure 1:**
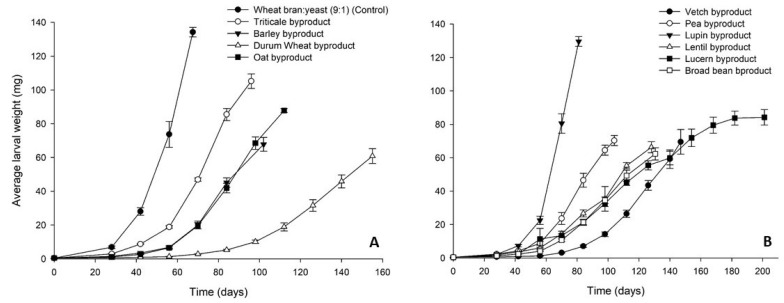
Average larval weight (mg) of *Tenebrio molitor* larvae reared on byproducts of the seed cleaning process of cereals (**A**) and legumes (**B**) or a mixture of wheat bran and yeast (9:1) (control). In all cases, values represent means ± SEM (n = 6).

The increase of *T. molitor* average larval weight on the different byproducts over time is graphically presented in Figure 1. Regarding the cereal byproducts (Figure 1A), triticale byproduct gave the best results close to the control (134 ± 3 mg), reaching a final average larval weight of 108 ± 6 mg, whereas the final average larval weight for the rest of the cereal byproducts ranged between 64 ± 9 (durum wheat byproduct) and 90 ± 5 mg (oat byproduct). Among the legume byproducts, lupin performed at best with a final average weight of *T. molitor* larvae of 130 ± 3 mg (Figure 1B). Lower final larval weights were recorded for the rest of the legume byproducts ranging between 63 ± 4 (broad bean byproduct) and 80 ± 5 (lucerne byproduct).

**Figure 2:**
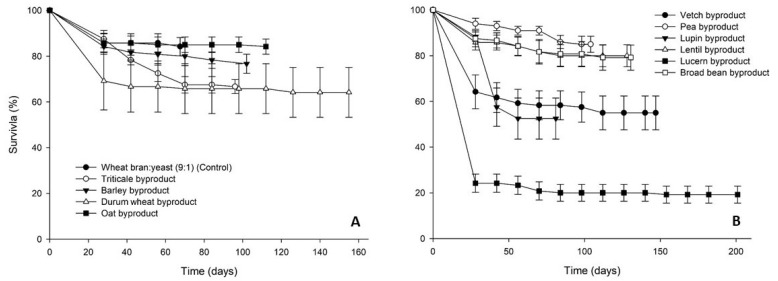
Survival rate (%) of *Tenebrio molitor* larvae reared on byproducts of the seed cleaning process of cereals (**A**) and legumes (**B**) or a mixture of wheat bran and yeast (9:1) (control). In all cases, values represent means ± SEM (n = 6).

Survival rates of *T. molitor* larvae varied considerably among dietary treatments (Figure 2). In the case of the cereal byproducts high final survival rates were recorded (>64%), the highest being noted for the oat byproduct (84%). Survival of larvae fed on legume byproducts followed a similar pattern (>52% survival), with the exception of lucerne byproduct, for which low survival rates (24%) were recorded already at the first evaluation time interval (week 4).

**Figure 3:**
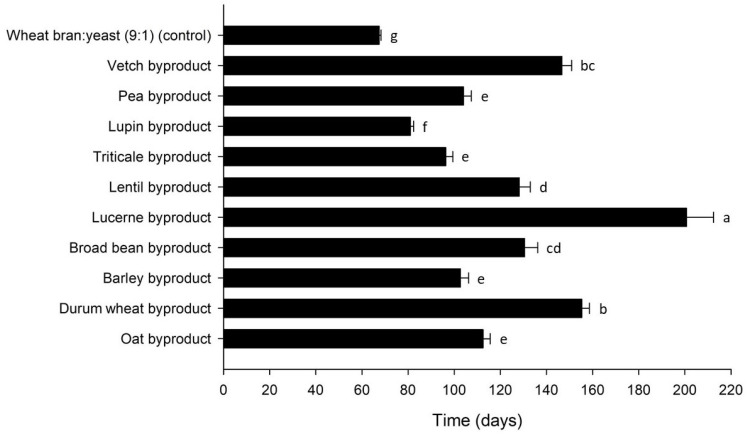
Development time (days) of *Tenebrio molitor* larvae reared on byproducts of the seed cleaning process of cereals and legumes or a mixture of wheat bran and yeast (9:1) (control). Bars followed by the same lowercase letter are not significantly different. In all cases, values represent means ± SEM (n = 6; df = 10; *p* = 0.05).

The larval development time for *T. molitor* was influenced by byproduct (Mantel–Cox χ^2^ = 189.7, df = 10, *p* < 0.001) and varied between 67 and 201 d over treatments (Figure 3). Apart from the control (67 ± 1 d), the shortest development times were observed for lupin (81 ± 1 d), triticale (96 ± 3 d) and barley byproduct (103 ± 4 d), whereas time to pupation was the longest for lucerne (201 ± 12 d). Shorter development times correlated with lower FCR (r = 0.660, *p* < 0.001) values, as well as with higher SGR (r = −0.886, *p* < 0.001) values. ECR values differed significantly among the byproducts tested (Table 2), depending on the byproduct cost and the respective FCR. There was a positive correlation between ECR and FCR (r = 0.577, *p* < 0.001), i.e., the higher the feed conversion efficiency for one byproduct, expressed with a lower FCR value, the lower the cost of using it for rearing.

### 3.2. Alphitobius diaperinus

**Figure 4:**
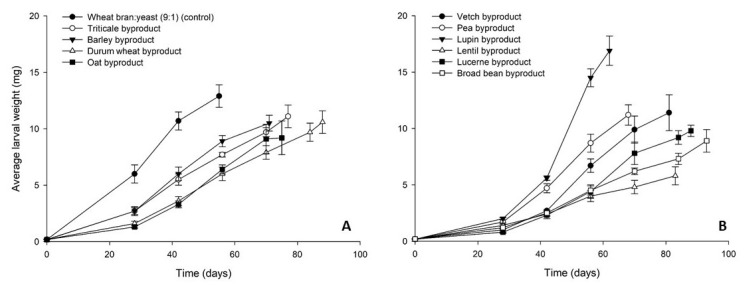
Average larval weight (mg) of *Alphitobius diaperinus* larvae reared on byproducts of the seed cleaning process of cereals (**A**) and legumes (**B**) or a mixture of wheat bran and yeast (9:1) (control). In all cases, values represent means ± SEM (n = 6).

**Figure 5:**
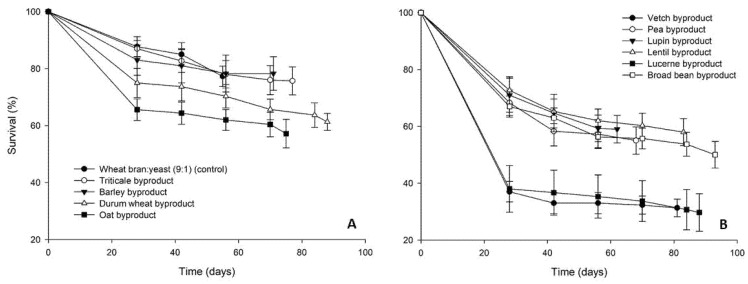
Survival rate (%) of *Alphitobius diaperinus* larvae reared on byproducts of the seed cleaning process of cereals (**A**) and legumes (**B**) or a mixture of wheat bran and yeast (9:1) (control). In all cases, values represent means ± SEM (n = 6).

The growth of *A. diaperinus* larvae on the tested byproducts and the control diet is presented in Figure 4. The highest final average larval weight was recorded for lupin byproduct (17 ± 1 mg) and was higher than control (13 ± 1 mg). The cereal byproducts clustered together, reaching a final individual larval weight between 10–11 mg (Figure 4A). Similar values were measured for the rest of the legume byproducts (9–12 mg), with the exception of lentil byproduct for which the lowest final larval weight was recorded (6 ± 1 mg) (Figure 4B). Larval survival rates at the end of the bioassay ranged between 50 and 78%, with the exception of lucerne (30%) and vetch byproduct (31%) (Figure 5).

**Figure 6:**
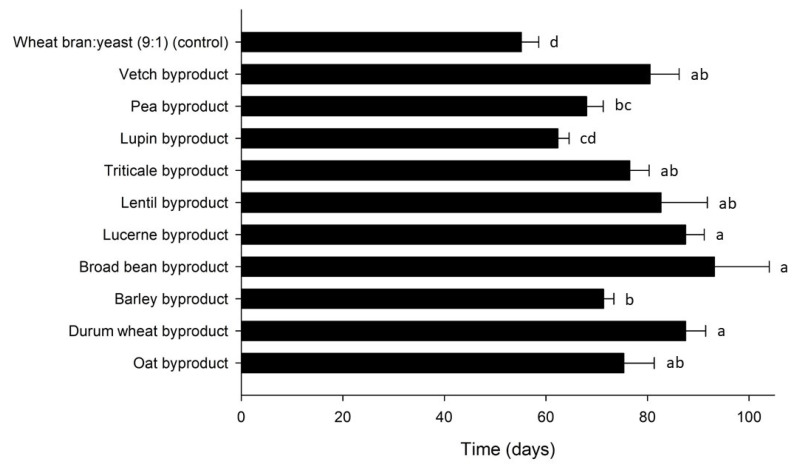
Development time (days) of *Alphitobius diaperinus* larvae reared on byproducts of the seed cleaning process of cereals and legumes or a mixture of wheat bran and yeast (9:1) (control). Bars followed by the same lowercase letter are not significantly different. In all cases, values represent means ± SEM (n = 6; df = 10; *p* = 0.05).

The larval development time for *A. diaperinus* was influenced by strain (Mantel–Cox χ^2^ = 66.4, df = 10, *p* < 0.001) and varied between 55 and 93 d over treatments (Figure 6). Larvae grew faster on the control diet (55 ± 3 d) and on the lupin byproduct (62 ± 2 d). Significant differences were observed among the FCR values calculated for the tested byproducts (Table 3). The lowest FCR values, corresponding to the highest feed conversion efficiencies, were recorded for the control diet (2.3), as well as the lupin (2.7), barley (4.8) and triticale byproduct (5.1). Similarly, the higher growth rates were obtained for the control diet (7.8% d^−1^) and for the lupin byproduct (7.3% d^−1^). There was a positive correlation between development time and FCR (r = 0.396, *p* = 0.001), and a negative correlation with SGR (r = −0.849, *p* = 0.001). The lowest ECR values were calculated for barley (667 € ton^−1^ larvae), triticale (719 € ton^−1^ larvae) and oat byproduct (755 € ton^−1^ larvae). As expected, lower ECR values, which correspond to a higher economic performance, correlated with lower FCR values (r = 0.730, *p* = 0.001), which are indicative of a higher feed conversion efficiency.

## 4. Discussion

In light of the results of the present study several byproducts of the seed cleaning process could be efficiently used as feeding substrates for *T. molitor* and *A. diaperinus* larvae. In the case of *T. molitor*, one cereal and one legume byproduct, i.e., the triticale and the lupin byproduct, gave the best results in terms of larval growth performance and development time. Interestingly, triticale and lupin byproduct had the lowest (8.5% DM) and highest protein content (33.5% DM), respectively, among the byproducts tested (Table 1). This indicates that, in contrast to what it is commonly believed that high-protein diets result in greater insect growth [30], other nutritional factors, such as fat content, amino acid profile, vitamins and minerals, may also substantially affect insect growth and development. This finding is in accordance with the results of a previous study, which evaluated the suitability of a number of cereal and legume commodities for *T. molitor* rearing [31]. In that study, larval growth did not always correlate well with the protein content of the commodity, indicating that the high protein content of a substrate, although important, does not always ensure increased larval growth, nor the opposite.

To our knowledge, this is the first report on the evaluation of triticale and lupin-based diets for *T. molitor* rearing. For a relative tenebrionid species, the red flour beetle, *Tribolium castaneum* (Herbst) (Coleoptera: Tenebrionidae), triticale was significantly better for larval growth than hard bread wheat and durum wheat, indicating the high nutritional value of this cereal and its potential as insect feeding substrate [32,33]. In a recent work studying the suitability of various byproducts of the agri-food industry, *T. molitor* larvae were offered feed mill byproducts (mainly broken cereal grains), which resemble the byproducts tested in our study [21]. Although the authors of that study do not provide information regarding the type of cereal grains that composed this particular byproduct to allow a direct comparison with the results of our study, feed mill byproducts supported *T. molitor* larvae development, however, larval mortality was higher and larval growth was lower than when fed with the control diet [21].

Ιn contrast to its closely related soya bean, which is one of the prevalent sources of plant proteins for food and feed applications, lupin is a rather underexploited nutrient source [34] with good nutritional traits [35]. Specifically, lupin grains have a higher protein and a lower carbohydrate content compared with other legumes, such as pea and chickpea [35], whereas their oil content is relatively low but of high quality [36]. For example, the protein content of dehulled lupin seeds is at the range of 39–55% of dry matter, which is comparable to that of soya bean [37]. Additionally, lupin is relatively free of antinutritional factors present in other legumes, which can positively affect its inclusion in food and feed products [35]. In the present study, the high protein content of the lupin byproduct (33.5%), which was the highest among the byproducts tested, may have boosted the *T. molitor* larval growth. These first results provide evidence that triticale and lupin byproducts could be used either singly or as ingredients of compound diets for *T. molitor* larvae rearing.

In the case of *A. diaperinus*, all cereal byproducts tested were suitable for rearing of its larvae, resulting in high final average larval weight (>10 mg). Data on the growth of *A. diaperinus* on cereal-based diets is rather limited. When fed with different cereal flours, i.e., wheat, barley, corn and rice flour, *A. diaperinus* growth was reduced compared to a mixture of whole meal flour:yeast used as control [38]. Moreover, differences were observed among the development rates on the various cereal flours tested [38]. Similar to *T. molitor*, among the legume byproducts tested the lupin byproduct was the best, in terms of larval growth and final weight at the end of the bioassay, indicating the potential of lupin byproducts for integration in the diets of *A. diaperinus* diets. In contrast, in lucerne high larval mortality rates were recorded for both *A. diaperinus* and *T. molitor* (20–30% final survival) and this effect may be attributed to specific components of this plant, such as saponins, which are known to have antinutritional properties [39,40]. Specifically for insects, it is suggested that saponins have also insecticidal activity, as the increase of lucerne saponin rates into an artificial diet of the European grape moth, *Lobesia botrana* Den and Schiff (Lepidoptera: Tortricidae), resulted in increased larval mortality [41]. Therefore, it could be suggested that the use of lucerne-related substrates should be avoided or extensively tested prior to inclusion in diets of *T. molitor* and *A. diaperinus*. This may be the case also for the vetch byproduct, in which low larval survival (31%) for *A. diaperinus* and reduced survival (55%) for *T. molitor* were recorded, as common vetch seeds also contain antinutritional factors which need to be removed or inactivated prior to utilization as feedstock [42].

For several of the tested byproducts, low FCR values were calculated, indicating a high feed utilization. For *T. molitor*, FCR values for pea, triticale, broad bean, oat and barley byproducts were similar to control and to the ones previously reported [15,24]. Similarly, high feed utilization efficiency was shown in the case of *A. diaperinus* for lupin, triticale, barley and oat byproduct. Published information on FCR values for *A. diaperinus* is scarce, however, low FCR values (3.0–3.2) have also been reported for high-protein content diets based on organic byproducts [24]. The economic efficiency of the use of a feeding substrate is highly important and heavily affects the economic performance of insect production. In the case of *T. molitor*, the lowest ECR was found for the triticale byproduct (361 €/ton), which was one of the cheapest byproducts tested (140 €/ton). In contrast, the high ECR calculated for the lupin byproduct (1418 €/ton), due to its high cost (300 €/ton), makes its utilization unrealistic and uneconomical for commercial and industrial rearing systems. What could be considered though, having in mind the good performance of *T. molitor* larvae on this substrate, is the low dietary inclusion of the lupin byproduct in compound diets for this species; however, this scenario has to be previously tested and validated. For *A. diaperinus*, the lowest ECR values were recorded for barley, triticale, oat and lupin byproducts at a range of 667–810 €/ton. Data on the cost of *A. diaperinus* rearing on different substrates is limited. When *A. diaperinus* larvae were fed with wheat middlings-based diets with different percentages of agro-food side-streams (rapeseed meal, rice bran, corn gluten and DDGS) the cost of larval production, expressed as €/Kg larvae, was considerably affected, either positively or negatively, by the byproduct inclusion [25]. However, since the calculated costs in that study are expressed as percentage of the cost of the control diet (wheat middlings), no direct comparisons are possible with the results of our study.

## 5. Conclusions

The results of the present study highlight the valorisation potential of several cereal and legume byproducts of the seed cleaning process for the rearing of larvae of *T. molitor* and *A. diaperinus*. Overall, the lupin and triticale byproducts efficiently supported complete larval development, i.e., from first instar to pupation, and gave the best results among the byproducts tested in terms of larval growth and survival, development time and feed utilization. Further studies should focus on the formulation of compound diets based on the best performing byproducts and the evaluation of their suitability as feeding substrates.

## Figures and Tables

**Table 1:** Dry matter (%), protein content (%DM) and cost (€/ton) of ten byproducts of the seed cleaning process of cereals and legumes and two control diet ingredients.

Byproduct	Dry Matter (%)	Protein (% DM)	Price (€/ton)
Wheat bran (control diet ingredient)	85.2	16.7	170
Vetch (*Vicia sativa*) byproduct	91.8	24.9	270
Pea (*Pisum sativum*) byproduct	91.9	28.2	220
Lupin (*Lupinus albus*) byproduct	93.3	33.5	300
Triticale (*Triticum* sp. x *Secale cereale*) byproduct	92.1	8.5	140
Lentil (*Lens culinaris*) byproduct	91.0	20.8	350
Lucerne *(Medicago sativa*) byproduct	95.8	13.3	100
Broad bean (*Vicia faba*) byproduct	91.0	27.3	220
Barley (*Hordeum vulgare*) byproduct	91.6	9.1	140
Durum wheat *(Triticum durum*) byproduct	91.0	11.0	170
Oat (*Avena sativa*) byproduct	90.4	12.3	140
Yeast (control diet ingredient)	97.9	50.0	8000

Crude protein = N × 6.25.

**Table 2:** Feed conversion ratio (FCR), specific growth rate (SGR, % day^−1^) and economic conversion ratio (ECR, € ton^−1^ larvae) of *Tenebrio molitor* larvae reared on byproducts of the seed cleaning process of cereals and legumes and a mixture of wheat bran and yeast (9:1) (control) (n = 6).

Substrate	FCR	SGR (% day^−1^)	ECR (€ ton^−1^ Larvae)
Wheat bran:yeast (9:1) (control)	2.3 ± 0.1 cd	8.6 ± 0.1 a	2211.2 ± 83.5 a
Vetch byproduct	7.9 ± 0.6 a	3.5 ± 0.1 d	2138.4 ± 148.8 a
Pea byproduct	1.7 ± 0.1 d	5.0 ± 0.2 bc	380.6 ± 30.4 d
Lupin byproduct	4.7 ± 1.0 abc	7.2 ± 0.1 ab	1418.4 ± 299.1 ab
Triticale byproduct	2.6 ± 0.3 cd	5.8 ± 0.2 ab	361.1 ± 40.4 d
Lentil byproduct	5.8 ± 0.4 ab	4.0 ± 0.1 cd	2020.7 ± 157.0 a
Lucerne byproduct	12.6 ± 3.0 a	2.7 ± 0.1 d	1260.5 ± 300.5 abc
Broad bean byproduct	2.9 ± 0.6 cd	3.9 ± 0.1 cd	629.4 ± 125.4 bcd
Barley byproduct	3.5 ± 0.2 bc	5.1 ± 0.1 abc	493.5 ± 34.0 cd
Durum wheat byproduct	7.6 ± 2.4 ab	3.3 ± 0.1 d	1284.8 ± 402.5 abc
Oat byproduct	3.1 ± 0.2 bc	4.8 ± 0.1 bc	428.0 ± 21.6 d

Within each column, means followed by the same lowercase letter are not significantly different. In all cases, values represent means ± SEM (n = 6; df = 10; *p* = 0.05).

**Table 3:** Feed conversion ratio (FCR), specific growth rate (SGR, % day^−1^) and economic conversion ratio (ECR, € ton^−1^ larvae) of *Alphitobius diaperinus* larvae reared on byproducts of the seed cleaning process of cereals and legumes and a mixture of wheat bran and yeast (9:1) (control) (n = 6).

Substrate	FCR	SGR (% day^−1^)	ECR (€ ton^−1^ Larvae)
Wheat bran:yeast (9:1) (control)	2.3 ± 0.2 e	7.8 ± 0.4 a	2185.0 ± 171.5 b
Vetch byproduct	15.2 ± 3.4 a	5.1 ± 0.2 cde	4092.3 ± 906.4 a
Pea byproduct	5.4 ± 0.7 bcd	6.2 ± 0.1 abc	1192.6 ± 152.0 bc
Lupin byproduct	2.7 ± 0.3 de	7.3 ± 0.2 ab	810.9 ± 84.5 cd
Triticale byproduct	5.1 ± 0.2 cde	5.4 ± 0.2 cde	719.2 ± 26.0 cd
Lentil byproduct	12.6 ± 1.2 a	4.5 ± 0.4 e	4410.2 ± 433.8 a
Lucerne byproduct	16.6 ± 5.5 a	4.6 ± 0.2 de	1663.7 ± 550.2 bc
Broad bean byproduct	10.7 ± 1.8 ab	4.3 ± 0.4 e	2361.9 ± 389.8 ab
Barley byproduct	4.8 ± 0.8 cde	5.7 ± 0.2 bcd	667.3 ± 117.5 d
Durum wheat byproduct	7.1 ± 0.5 abc	4.6 ± 0.1 e	1202.9 ± 81.2 bc
Oat byproduct	5.4 ± 0.4 bcde	5.4 ± 0.3 cde	755.3 ± 55.0 cd

Within each column, means followed by the same lowercase letter are not significantly different. In all cases, values represent means ± SEM (n = 6; df = 10; *p* = 0.05).
